# Virtual Experiments Enable Exploring and Challenging Explanatory Mechanisms of Immune-Mediated P450 Down-Regulation

**DOI:** 10.1371/journal.pone.0155855

**Published:** 2016-05-26

**Authors:** Brenden K. Petersen, Glen E. P. Ropella, C. Anthony Hunt

**Affiliations:** 1 UCSF/UCB Joint Graduate Group in Bioengineering, University of California, Berkeley, California, United States of America; 2 Tempus Dictum, Inc., Portland, Oregon, United States of America; 3 Department of Bioengineering and Therapeutic Sciences, University of California San Francisco, San Francisco, California, United States of America; Koc University, TURKEY

## Abstract

Hepatic cytochrome P450 levels are down-regulated during inflammatory disease states, which can cause changes in downstream drug metabolism and hepatotoxicity. Long-term, we seek sufficient new insight into P450-regulating mechanisms to correctly anticipate how an individual’s P450 expressions will respond when health and/or therapeutic interventions change. To date, improving explanatory mechanistic insight relies on knowledge gleaned from in vitro, in vivo, and clinical experiments augmented by case reports. We are working to improve that reality by developing means to undertake scientifically useful virtual experiments. So doing requires translating an accepted theory of immune system influence on P450 regulation into a computational model, and then challenging the model via in silico experiments. We build upon two existing agent-based models—an in silico hepatocyte culture and an in silico liver—capable of exploring and challenging concrete mechanistic hypotheses. We instantiate an in silico version of this hypothesis: in response to lipopolysaccharide, Kupffer cells down-regulate hepatic P450 levels via inflammatory cytokines, thus leading to a reduction in metabolic capacity. We achieve multiple in vitro and in vivo validation targets gathered from five wet-lab experiments, including a lipopolysaccharide-cytokine dose-response curve, time-course P450 down-regulation, and changes in several different measures of drug clearance spanning three drugs: acetaminophen, antipyrine, and chlorzoxazone. Along the way to achieving validation targets, various aspects of each model are falsified and subsequently refined. This iterative process of falsification-refinement-validation leads to biomimetic yet parsimonious mechanisms, which can provide explanatory insight into how, where, and when various features are generated. We argue that as models such as these are incrementally improved through multiple rounds of mechanistic falsification and validation, we will generate virtual systems that embody deeper credible, actionable, explanatory insight into immune system-drug metabolism interactions within individuals.

## Introduction

Hepatic cytochrome P450 (P450) is the major family of drug-metabolizing enzymes in the liver. Changes in P450 levels are common among many disease states, giving rise to the concern that a patient may experience an imbalance in drug exposure when the disease alters P450 levels and downstream drug metabolism. Though a small subset of P450s are induced by inflammation, most inflammatory states down-regulate hepatic P450, reducing drug clearance and elevating plasma drug levels, thus increasing the risk of adverse effects—especially for low therapeutic index drugs [1,2]. P450 down-regulation can also protect against toxicity caused by reactive metabolites [2,3]. For example, pretreatment with an inflammatory stimulus protects against acetaminophen-induced hepatotoxicity [4].

Inflammatory-induced P450 down-regulation is mediated by proinflammatory cytokines, including interleukin (IL)-1β, IL-6, and tumor necrosis factor-α (TNF-α), that specifically regulate different yet overlapping subsets of P450s in both humans and rats [5,6]. Many of these cytokines are derived from Kupffer cells. While some cytokines down-regulate P450 in primary hepatocytes cultures, others are dependent upon the presence of Kupffer cells [7]. Kupffer cells can be activated by bacterial endotoxin (lipopolysachharide, LPS). An LPS stimulus causes Kupffer cells to release proinflammatory cytokines, triggering P450 down-regulation. For more information, we refer the reader to four reviews on immune-mediated P450 down-regulation [1–3,8].

Long-term, we seek sufficient new insight into P450-regulating mechanisms to correctly anticipate how an individual’s P450 expressions will respond when health and/or therapeutic interventions change. To date, improving explanatory mechanistic insight relies on knowledge gleaned from in vitro, in vivo, and clinical experiments augmented by case reports. We are working to improve that reality by developing means to undertake scientifically useful virtual experiments [9,10]. To be scientifically useful, the computational models employed must demonstrate credibility, in part by meeting demanding representational requirements. For example, not only must the simulated phenomena be quantitatively similar to available wet-lab data, but the software mechanisms—the actual events occurring during execution—should also be demonstrably biomimetic. Making key aspects of both model and experiment increasingly analogous to past or future real-world counterparts further enhances credibility. Advances in agent-based modeling and simulation (M&S) methods have now made it feasible to begin achieving such requirements [11]. We report further progress.

A prerequisite for achieving our objective is to translate a currently accepted theory of immune system influence on P450 regulation (such as that cited above) into a computational model, and then challenge the model via in silico experiments to generate phenomena that are measurably similar to preselected data reported in the literature. An agent-based model’s mechanisms will be a concretized hypothesis: these components interacting in these spaces, following these rules, under these constraints will, upon execution, produce material system changes, which when measured will be within (for example) ± 10% of the corresponding wet-lab values. When the initial hypothesis fails—and it almost always does—we posit explanations, revise both hypothesis and model, and repeat the challenge. In this report, we begin with two existing agent-based models—an in silico hepatocyte culture (ISHC) [12] and an in silico liver (ISL) [13,14]—that have already achieved many validation targets related to drug metabolism and hepatotoxicity. We then repurpose both models to support the additional use case of exploring mechanisms related to immune system involvement in the liver. Specifically, we instantiate and challenge the following mechanistic hypothesis in the ISHC and ISL: in response to LPS, Kupffer cells down-regulate hepatic P450 levels via inflammatory cytokines, thus leading to a reduction in metabolic capacity.

Knowledge about interactions among hepatic P450-regulating mechanisms and immune system components comes from both in vitro and in vivo experiments. An essential, demanding requirement herein is thus that the same mechanism components must be utilized in models simulating in vitro and in vivo environments. We report quantitative validation evidence supporting an in vitro (ISHC) and in vivo model (ISL). By employing modularization and integration techniques [12], we enable reuse of components and mechanisms between ISHC and ISL. We conceptually deconstruct the above hypothesis into three “stages” and achieve degrees of validation for each stage: 1) Kupffer cells produce cytokines upon LPS stimulus, 2) cytokines down-regulate hepatic P450 levels, and 3) P450 down-regulation reduces drug clearance. We achieve multiple in vitro and in vivo validation targets gathered from five wet-lab experiments, including a LPS-cytokine dose-response curve, time-course P450 down-regulation, and changes in several different measures of drug clearance spanning three drugs: acetaminophen (APAP), antipyrine (ANT), and chlorzoxazone (CZN). During the validation process, we falsify several mechanistic details and other ISHC and ISL components, to which we respond by iteratively refining model aspects until validation targets are achieved. This iterative process of falsification-refinement-validation ensures that model components are increasingly biomimetic yet parsimonious. Such models are perpetual works in progress. We argue that as these models are incrementally improved through multiple future rounds of mechanistic challenge and validation against an expanding set of measured attributes, we will generate virtual systems that embody deeper credible, actionable, explanatory insight into immune system-drug metabolism interactions within individuals.

## Methods

To avoid ambiguity between in silico components and their referent biological counterpart, we use small caps when referring to the former, e.g. hepatocyte. Parameter names are italicized. Analogs were written in Java, utilizing the MASON multi-agent simulation toolkit [15]. In silico experiments were run using 2 or 16 node virtual machines on Google Compute Engine, running 64-bit Debian 7. For longer simulations, Monte Carlo trials were run in parallel.

### Synthetic and agent-based modeling

Exploring the causal mechanisms underlying P450 down-regulation can be facilitated using synthetic M&S methods. Synthetic M&S is a developing method that fundamentally differs from conventional equation-based models (i.e. physiologically based pharmacokinetic/pharmacodynamic models) in several ways. In synthetic M&S, autonomous software objects representing components such as drugs, enzymes, cells, and tissues are plugged together to form a coherent whole. The resulting multi-scale model is called a *biomimetic analog*. Analog components have several mechanisms—composed of rules, equations, and/or other operating principles—that specify how to interact with other components. At the software level, analog components are dynamic data structures, containing state information that changes as the simulation evolves; analog mechanisms are sets of governing logic that manipulate component state information.

An analog is an experimental apparatus that can be used to challenge mechanistic hypotheses about referent phenomena. When an analog is executed, its components and mechanisms are instantiated—represented by a concrete instance—in silico [16]. The analog produces phenomena, some of which are intended to mimic those of its referent system. If analog phenomena are acceptably similar to wet-lab and/or clinical validation data, we support the following hypothesis: events that transpired in silico may have biological counterparts. Thus, a validated analog stands as a challengeable theory about mechanistic events that may have occurred in the referent system. Alternative analog mechanisms can be tested in parallel. As we continue to refine and expand surviving analog components and mechanisms to mimic an ever-increasing set of validation data, we grow increasingly confident that the analog behaves analogously to the referent under specified conditions.

The main objective for synthetic M&S is to build better working hypotheses about the mechanisms of interest. Thus, an important requirement is that analogs are suitable for virtual experimentation and, therefore, hypothesis testing. An experiment on an analog is an in silico experiment, precisely analogous to a wet-lab or clinical experiment. As such, analogs include components and mechanisms that map to concrete, relevant aspects of a wet-lab or clinical experiment, including biological components (e.g. hepatocytes), wet-lab and/or clinical environments (e.g. in vitro, in vivo), experimental procedures (e.g. intravenous drug injection), and measurements (e.g. plasma concentration profile). Thus, a virtual experiment using an analog such as the ISL is not simply a model of (say) “drug metabolism” or some other particular biological process. Rather, an instantiated ISL is a concrete software analog of (say) a whole rat intravenously injected with 20 mg/kg acetaminophen and measured at 15 min intervals via external jugular cannulation. An advantage of such virtual experimentation is that variations of a single analog can be used to mimic a wide range of experimental protocols, treatments, disease states, and measurements [10]. When new use cases require including new aspects of the referent experiment (e.g. an alternative hypothesized mechanism, a new in silico measurement, a different experimental protocol, or an altered disease state), it is straightforward to add that level of detail.

There are several differences between synthetic M&S and traditional inductive methods, which arise largely from differences in model use case and goals. Whereas synthetic M&S focus on challenging concrete mechanisms, inductive approaches (e.g. equation-based, continuous mathematics models) typically employ equations that describe patterns in data and are used to make precise predictions about future patterns in data. For these uses, conventional methods are unsurpassed. Conventional models can also be used to challenge mechanistic hypotheses (for example, by changing parameters that map to different routes of administration); however, it is more difficult to challenge a series of alternative mechanisms, possibly at different levels of granularity. While it is straightforward for synthetic analogs to change mechanistic detail or challenge multiple mechanisms in parallel, conventional equation-based models often require significant model re-engineering—or a completely new model—when adding new variables, changing granularity, or switching use cases. Validation targets herein span multiple wet-lab platforms (in vitro and in vivo) and measurements types (e.g. three different measures of drug clearance); thus, synthetic approaches are more appropriate for purposes herein. Other key features of the similarities and differences between synthetic M&S and conventional inductive models have been detailed elsewhere [11,16,17].

Note that it would be straightforward to employ traditional modeling approaches (e.g. pharmacokinetic/pharmacodynamic modeling) to describe the biological processes involved in the immune-mediated P450 down-regulation pathway. Given sufficient P450 isozyme-specific validation data, such models may be useful, for example, in precisely predicting levels of P450 down-regulation following inflammatory stimulus. However, the ability to explore, challenge, and refine mechanistic hypotheses related to P450 down-regulation is better suited for synthetic analogs. Further, other pharmacologically relevant phenomena are difficult or problematic to describe using traditional multi-compartment models—e.g. liver zonation (i.e. lobule location-dependent effects) or leukocyte recruitment and localization—but are straightforward to reproduce using spatially explicit synthetic analogs. Since long-term objectives include exploring mechanisms of such attributes, synthetic analogs are the more appropriate choice.

Agent-based modeling is one model type that can be used to achieve the above synthetic M&S goals. An agent-based model contains agents, which are software objects capable of scheduling their own events [11]. Each agent senses, interacts with, and is a part of a virtual environment. They each follow a set of governing rules or operating principles, the logic of which is specified by the modeler. In biological M&S, agents typically map to (represent) biological components (e.g. cells), and their operating principles map to cellular interactions and processes. We refer the reader to [11] for an in-depth review of the role of synthetic M&S utilizing agent-based modeling in pharmaceutical research.

### Iterative refinement protocol

**Fig 1:**
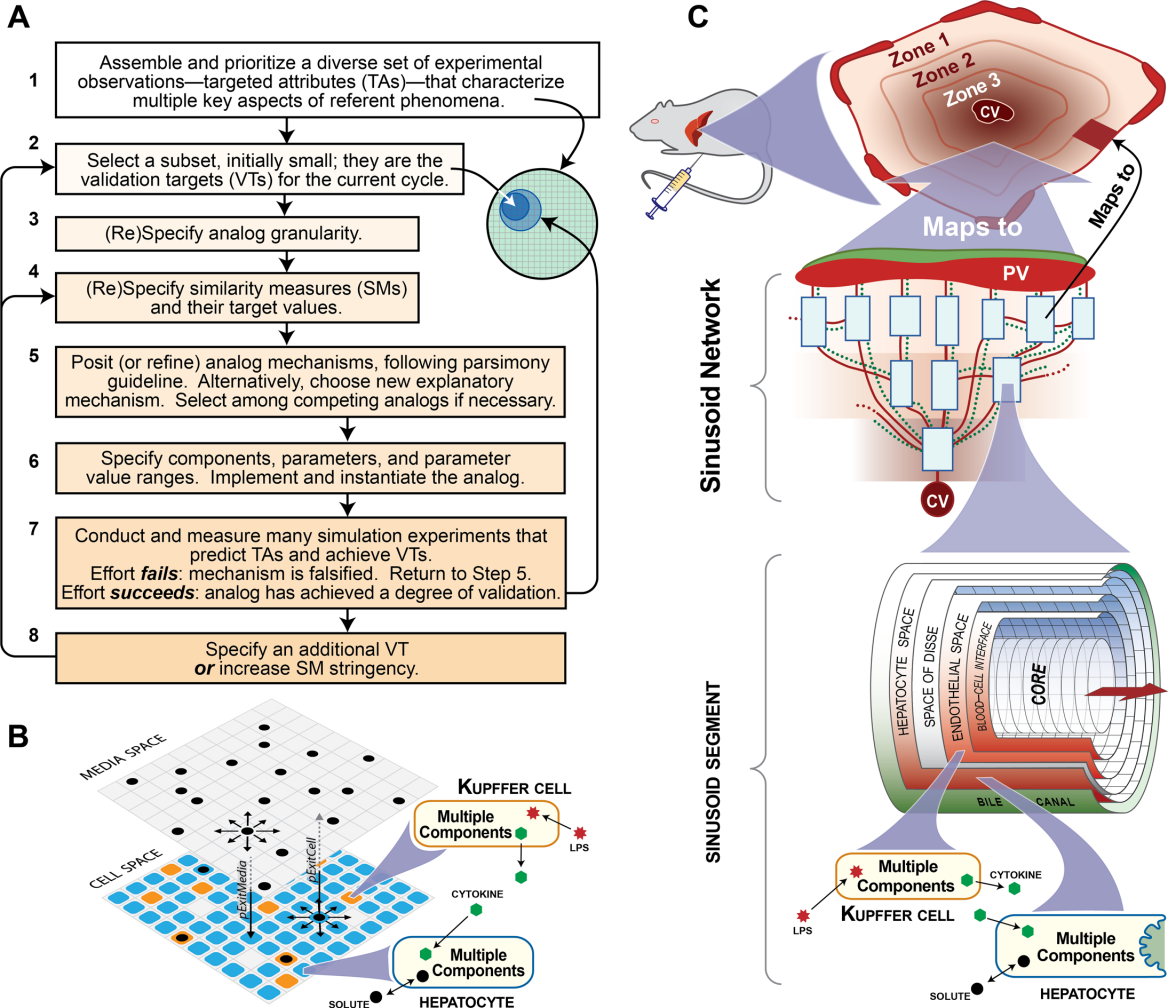
Analog methods and structure. **A.** An iterative protocol for refining biomimetic analogs. **B.** Key features of ISHC structure. The ISHC contains two grids: cell space and media space (only portions of each grid are shown). Solutes can move laterally within a grid, or between cell space and media space, subject to the parameters *pExitMedia* and *pExitCell*. Select hepatocyte and Kupffer cell components are shown. **C.** Key features of ISL structure. There are two major components: body and lobule. Solutes are injected into body, where they distribute to the portal vein (PV) of the lobule. Solutes percolate through a network of sinusoid segments (SS) toward the central vein (CV), from which they return to body. Solutes can also move radially within a sinusoid segment through various spaces. Select hepatocyte and Kupffer cell components are shown.

A core principle of biomimetic M&S is to develop software models that provide increasingly credible mechanistic explanations of their referent complex biological processes. Such increases require the strategic use of parsimony. Abstract models will be falsified when challenged against specific validation data. When a model fails, the appropriate response is to refine it: either by increasing complexity (e.g. by adding parameters or switching to finer-grained components) or testing alternative mechanisms and/or structures before repeating the challenge. In support of this core principle, our model development process is governed by an iterative refinement protocol (IRP), a scientific method for falsifying, refining, and validating multi-scale biomimetic analogs. Described in Fig 1A, the IRP focuses on model falsification and subsequent refinement, following a strict parsimony guideline. The goal of stepping through an IRP cycle is to formulate a mechanistic hypothesis by meeting a set of validation targets. When *prespecified* similarity criteria are attained for a targeted attribute, we have achieved a validation target. Similarity criteria can range from qualitative (e.g. event *X* occurs before event *Y*) to quantitative (e.g. in silico points fall within ± 1 standard deviation of the corresponding wet-lab value). An analog mechanism is falsified when it cannot achieve a given validation target, either because the target phenomenon cannot be generated or we fail to find parameter set values that satisfy all similarity criteria.

When a mechanism is falsified, we specify a revision—a new hypothesis—which usually involves adding and/or revising analog components and/or mechanisms, perhaps at a finer level of granularity. To be scientifically meaningful, refinements should be parsimonious: we seek the simplest analog that still (“just barely”) achieves validation targets. If an analog mechanism is too simple for a given set of validation targets, it will be falsified, and iterative refinement will lead to a new mechanism. However, for an analog that is too complex (over-mechanized, analogous to an equation-based model being overparameterized), we waste computational resources, and it becomes difficult to identify where and why a future mechanism is falsified, rendering reengineering problematic.

### ISL and ISHC use cases

Both the ISL and ISHC are biomimetic analogs used to challenge mechanistic hypotheses related to drug metabolism and hepatotoxicity. As primarily exploratory devices, their focus shifts away from precise prediction of pharmacological values—for which traditional continuous mathematics and/or statistical methods are unsurpassed—and instead toward developing flexible methods to simulate mechanistic scenarios. It is easy to add mechanistic details that improve apparent realism of an ABM (e.g. [18]). However, to retain scientific usefulness, we strive to keep analog mechanisms parsimonious.

The ISL simulates drug clearance and hepatotoxicity experiments in an in vivo setting. It is a highly flexible platform that can mimic many experimental use cases. One use case configuration maps to a portion of an in situ isolated, perfused rat liver, in which simulations mimic the multiple indicator dilution method to measure hepatic outflow profile [19]. This iteration of the ISL includes a whole-rat configuration that can simulate either oral or intravenous drug administration. Measurements on the ISL range from measures of metabolism (e.g. hepatic outflow profile, extraction ratio, and intrinsic clearance) to necrosis (e.g. the time and location of cell death events). The current use case expands the ISL to include analogs of immune system components and mechanisms; experiments are conducted to test the entire P450 down-regulation pathway from LPS stimulus to decreased clearance. In particular, ISL simulations here mimic several wet-lab validation experiments in which rats are pretreated with LPS before administering drug (see Results for experiment details) [20–22]. Measurements include changes in drug clearance and P450 amounts.

The ISHC simulates drug clearance and hepatotoxicity experiments in an in vitro setting. A simulation maps to a portion of a monolayer culture of isolated rat hepatocytes and/or Kupffer cells. While much simpler than the ISL, the ISHC is useful for mimicking small parts of the immune-mediated P450 down-regulation pathway. In particular, the current ISHC use cases mimic wet-lab experiments in which cultured hepatocytes produce cytokine in response to LPS [23] and cultured Kupffer cells down-regulate P450 in response to cytokine (see Results for experiment details) [24]. Measurements include a LPS-cytokine dose-response curve and time-course enzyme levels.

### Selection of drugs and similarity criteria

We selected validation data for three drugs (APAP, ANT, and CZN) having different physiochemical properties and spanning a ~4-fold range in half-life in rats. Additional drugs can be added to the validation dataset to further improve model confidence; we selected three to demonstrate P450 down-regulation mechanisms. Indeed, earlier versions of the ISHC and ISL have been used for different use cases spanning additional drugs: see [12,25]. When introducing new analog drug objects into the simulation, we would first refer to literature reports of fraction metabolized. For the three chosen drugs, fraction metabolized in rats is nearly 100% [26–28]. We would next consider what fraction of metabolic clearance is due to P450 isoforms—where our attention is focused. For ANT and CZN, that is nearly 100% [27,28]; it is only a minor pathway (though important to toxicity) for APAP, dominated by cytochromes P450 2E1 and 1A2 [26,29]. Given fraction metabolized via specific enzymes, we refine the ISL and ISHC to include enzyme- and cytokine-specific functions (see “Mechanism granularity and flexibility”).

The choice of similarity criteria is governed by model use case. For example, a model intended to be used for precise prediction requires more stringent similarity criteria than a model used to explore explanatory mechanisms. When possible, similarity criteria were chosen to reflect variability in wet-lab measurements. Thus, a good starting point for moderately stringent similarity criteria is that in silico values fall within ± 1 standard deviation of the corresponding wet-lab values. When standard deviation is not given or cannot be determined from the validation data, an alternative is to specify an arbitrary percentage range. In these cases, we stipulated a range of ± 25%, which we found to be reflective of most related, reported standard deviation values. As an exception, we chose a range of ± 10% for ANT half-life to better reflect the smaller standard deviations of half-lives measures for other drugs. For validation targets that include many values (e.g. time-course data), we stipulate that at least 50% of in silico values must fall within the prespecified range. So doing prevents present but statistically insignificant abnormalities in a plot’s microstructure from prohibiting model development in early stages of validation and reduces the risk of overfitting to one particular dataset. For an example of such irregularities in microstructure, validation data from [20] at 80 min shows a statistically insignificant nonlinear dip in the semi-logarithmic drug disappearance curve for APAP, which is not found in similar studies (e.g. [30,31]).

### ISL and ISHC components

Upon execution, time advances through simulation cycles. Agents are scheduled to execute once each simulation cycle, with the exception of some events that are separately scheduled. Components common to both the ISL and ISHC include solutes, enzymes, and cells. Solutes are mobile objects that map to a group of small molecules. They percolate through spaces, influenced by various flow parameters (see Table 1). Solutes can have any number of properties that are specified offline as part of the parameter list (see Table 2). For example, only “bindable solutes” (solutes for which the Boolean parameter *bindable* is true) may bind to enzymes. Each solute is assigned a type that controls how other objects may interact with it. Herein, solute types include lps, cytokine, drug (apap, ant, or czn), and metabolite (apap-metabolite, ant-metabolite, or czn-metabolite); only the three drug types are bindable. For current use cases, only a single cytokine type was necessary; thus, cytokine maps to the set of all cytokines that may cause hepatic P450 down-regulation. However, when required in future use cases, cytokine may be replaced with objects that map to specific cytokines (e.g. interleukin-1), each with unique parameter values. There are no restrictions on the type or amount of information that can be “attached” to each solute; additional properties are added based on use case and as additional validation targets are achieved.

**Table 1:** ISL and ISHC parameters descriptions and values for validating experiments.

Parameter name	Type/Range	ISHC value(s)	ISL value(s)	Description
**Simulation control parameters**				
*cycleLimit*	positive integer	{2880; 1440}	variable	Number of simulation cycles after which simulation stops.
*monteCarloTrials*	positive integer	16	16	Number of Monte Carlo trials to execute.
**Dosing parameters**				
*doseTime* (solute type)	positive integer	{1 (lps); 1 (cytokine)}	{1 (drug); 1 (lps), 86401 (drug)}	Simulation cycle at which to administer solute dose. Multiple doses are separated by commas.
*dosage*	positive integer	{variable; 2000}	{62500; 125000}	Number of solute objects to administer each dose.
**Binding handler parameters**				
*pBind*	[0.0, 1.0]	N/A	0.25	Base probability for an unbound solute to bind to an unbound enzyme.
*bindCycles*	positive integer	N/A	10	Number of a simulation cycles a bound solute remains bound to an enzyme.
*bindExponent*	positive, real	N/A	see Table 3	Exponent that controls the degree to which increasing the number of bound enzymes decreases the probability of a binding event.
*bindable*	Boolean	see Table 2	see Table 2	If TRUE, this solute may bind to an enzyme.
*acceptedSolutes*	list	see Table 3	see Table 3	List of solute types that can bind to this enzyme.
**Metabolism handler parameters**				
*pMetabolize*	[0.0, 1.0]	N/A	see Table 2	Probability that a bound solute is metabolized by its bound enzyme.
*metabolic*	Boolean	see Table 3	see Table 3	If TRUE, this enzyme may metabolize bound solutes.
*metabolicProduct*	list	see Table 2	see Table 2	List of metabolites (if any) to produce upon metabolism.
**Inflammation handler parameters**				
*inflammatoryThreshold*	non-negative integer	{1; N/A}	3	Threshold number of inflammatory stimuli above which a cytokine may be produced.
*cytokineThreshold*	non-negative integer	{2; N/A}	2	Threshold number of cytokines above which no more cytokines may be produced.
*cytokineExponent*	positive, real	{3.0; N/A}	3.0	Exponent that controls the degree to which increasing inflammatory stimuli increases *pCytokine*.
*inflammatory*	Boolean	see Table 2	see Table 2	If TRUE, this solute may cause Kupffer cells to produce cytokines.
**Down-regulation handler parameters**				
*pRemove*	[0.0, 1.0]	{N/A; 0.05}	see Table 3	Probability that a cytokine removes an enzyme.
*delay*	non-negative integer	{N/A; 30}	600	Number of simulation cycles to delay before an enzyme scheduled for removal is actually removed.
*pReplenish*	[0.0, 1.0]	{N/A; 0.007}	0.0001	Probability that an enzyme is created when there are no cytokines, the removal queue is empty, and there are fewer than the starting number of enzyme.
*downRegulated*	Boolean	see Table 3	see Table 3	If TRUE, this enzyme may be down-regulated by cytokines.
**Degradation handler parameters**				
*pDegrade*	[0.0, 1.0]	see Table 2	see Table 2	Probability that an unbound solute is degraded.
**Cell parameters**				
*enzymesPerCellMin*	non-negative integer	4	4	The minimum number of enzyme of each type to create in each cell at the start of the simulation.
*enzymesPerCellMax*	non-negative integer	8	8	The maximum number of enzyme of each type to create in each cell at the start of the simulation.
*ecDensity*	[0.0, 1.0]	N/A	0.66	Probability that a grid point in endothelial space contains an endothelial cell.
*kcDensity*	[0.0, 1.0]	{1.0; 0.0}	0.33	Probability that a grid point in cell space (ISHC) or endothelial space (ISL) contains a Kupffer Cell.
*hepDensity*	[0.0, 1.0]	{0.0; 1.0}	0.9	Probability that a grid point in cell space (ISHC) or hepatocyte space (ISL) contains a hepatocyte.
*expressingCellTypes*	list	see Table 3	see Table 3	List of which cell types contain this enzyme type.
**Solute flow parameters**				
*sampleRatio*	[0.0, 1.0]	N/A	0.00115	Base fraction of solutes in body that are transferred to portal vein each simulation cycle.
*sampleRatioFactor*	positive, real	N/A	see Table 2	The fraction of solutes in body that are transferred to portal vein each simulation cycle is multiplied by this factor.
*V*_*d*,*change*_	positive, real	N/A	see Table 2	The fraction of solutes in body that are transferred to portal vein each simulation cycle is reduced by this factor in LPS experiments. Further, the original amount of administered solutes in reduced by this factor in LPS experiments.
*forwardBias*	[0.0,1.0]	N/A	0.2	Weight given to forward movement of a solute object, modifying the otherwise "Brownian" motion.
*flowRate*	positive integer	N/A	2	Number of grid points solute in the core is moved forward each simulation cycle.
*lateralBias*	[0.0, 1.0]	N/A	0.6	Weight given to lateral movement of a solute object, modifying the otherwise "Brownian" motion.
*pExitMedia*	[0.0, 1.0]	see Table 2	N/A	Probability that a solute can move from media space to cell space.
*pExitCell*	[0.0, 1.0]	see Table 2	N/A	Probability that a solute can move from cell space to media space.

**Fig 2:**
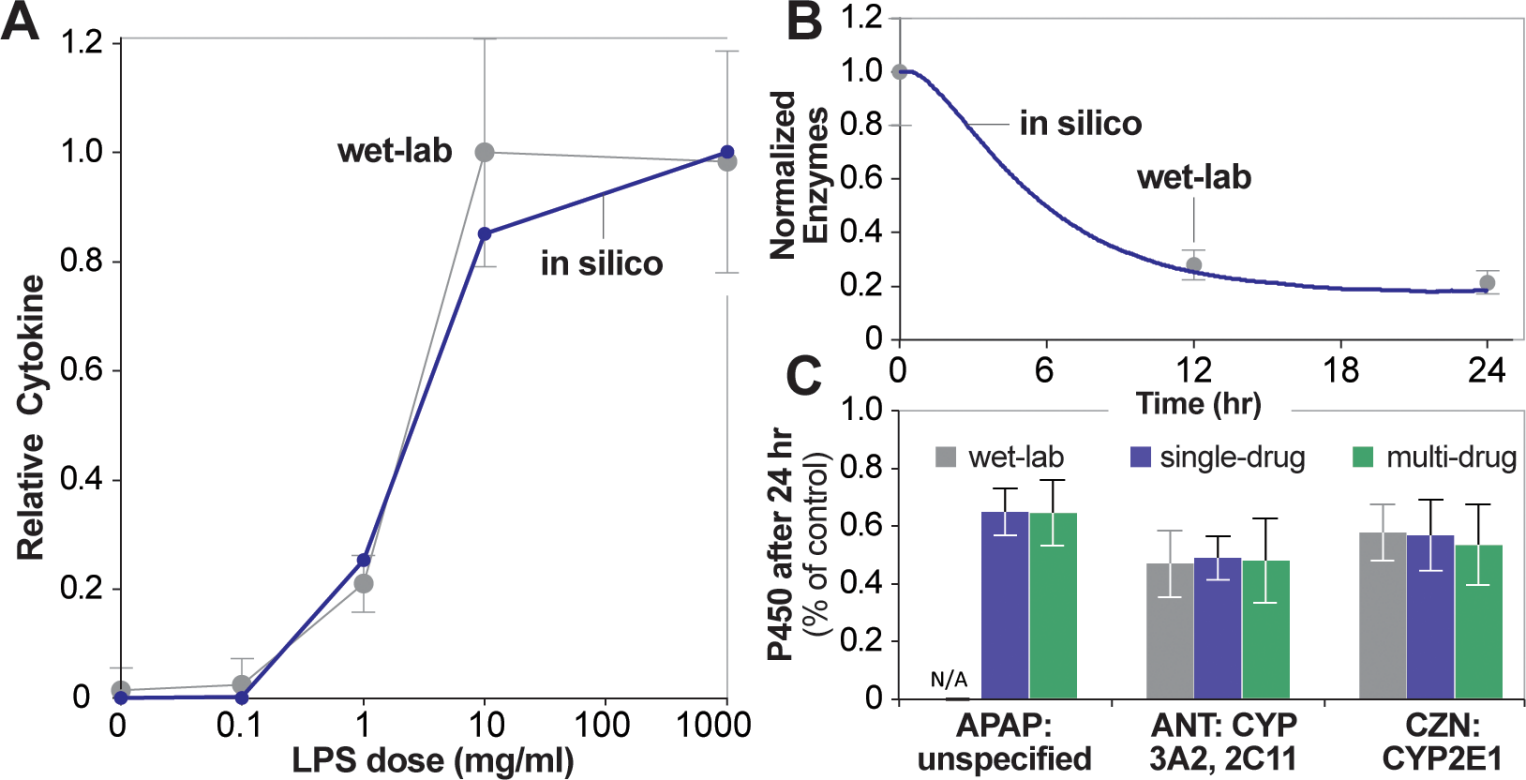
Validation targets for LPS treatment, before or without drug administration. **A.** Dose-response curve between LPS stimulus and normalized cytokine response. Values were measured after 48 hr (2,880 simulation cycles). Error bars: wet-lab standard deviation. In silico points are averages of 16 Monte Carlo trials. Wet-lab values are from [23]. **B.** Time-course levels of enzymes, normalized by the starting value. Error bars: wet-lab standard deviation. In silico points are averages of 16 Monte Carlo trials. Wet-lab values are from [24]. **C.** Wet-lab and in silico P450 levels relative to control values. Wet-lab values are relative measures of CYP3A2 (ANT) or CYP2E1 (CZN). In silico values are relative measures of the respective enzyme type. Error bars: standard deviation. Wet-lab values are from [20] (APAP), [21] (ANT), and [22] (CZN). Note [20] did not provide P450 data for APAP, but we included in silico values for comparison.

When ISHC parameter values differ between dose-response experiments (used to generate Fig 2A) and time-course experiments (used to generate Fig 2B), the different values are shown in brackets separated by semicolons: e.g. {dose-response value; time-course value}. Similarly, when ISL parameter values differ between control and LPS experiments, the different values are shown in brackets separated by semicolons: e.g. {control value; LPS value}. “N/A” values denote that the parameter is either not included in that simulation (e.g. an ISHC-specific parameter in an ISL simulation) or is not relevant in that simulation (e.g. metabolism handler parameters in ISHC simulations without drug). If a value states “see Table 2,” that parameter differs based on solute type; see Table 2 for solute-specific values. Similarly, if a value states “see Table 3,” that parameter differs based on enzyme type; see Table 3 for enzyme-specific values. The ISL values for *cycleLimit* are variable: for control experiments, the values are 6000 (for apap), 18000 (for ant), and 7200 (for czn); for LPS experiments, the values are 92400 (for apap), 104400 (for ant), and 93600 (for czn). The ISHC dose-response values for *dosage* are also variable: the dose-response curve was measured at 0, 70, 700, 7000, and 700000 lps objects.

**Table 2:** Solute-specific parameter values for validating experiments.

**Solute type**	***bindable***	***inflammatory***	***pMetabolize***	***metabolicProduct***	***pDegrade***	***sampleRatioFactor***	***V***_***d*,*change***_
**ISL simulations**							
apap	TRUE	FALSE	[0.35, 0.95]	apap-metabolite	N/A	1.0	N/A
ant	TRUE	FALSE	[0.35, 0.95]	ant-metabolite	N/A	0.26	N/A
czn	TRUE	FALSE	[0.35, 0.95]	czn-metabolite	N/A	0.52	1.69
lps	FALSE	TRUE	N/A	N/A	0.0005	N/A	N/A
cytokine	FALSE	FALSE	N/A	N/A	0.002	N/A	N/A
apap-metabolite	FALSE	FALSE	N/A	N/A	N/A	N/A	N/A
ant-metabolite	FALSE	FALSE	N/A	N/A	N/A	N/A	N/A
czn-metabolite	FALSE	FALSE	N/A	N/A	N/A	N/A	N/A
**Solute type**	***bindable***	***inflammatory***	***pMetabolize***	***metabolicProduct***	***pDegrade***	***pExitCell***	***pExitMedia***
**ISHC simulations**							
lps	FALSE	TRUE	N/A	N/A	N/A	1	{0.5; 0.1}
cytokine	FALSE	FALSE	N/A	N/A	{0.01; 0.002}	{0.01; 0.2}	0.02

When ISHC parameter values differ between dose-response experiments (used to generate Fig 2A) and time-course experiments (used to generate Fig 2B), the different values are shown in brackets separated by semicolons: e.g. {dose-response value; time-course value}. “N/A” values denote that the parameter is not relevant in that simulation (e.g. metabolism handler parameters in ISHC simulations without drug). Note ISL *pMetabolize* values are given as a range; cells nearest the portal vein exhibit the minimum value, cells nearest the central vein exhibit the maximum value, and the value is linearly interpolated for cells in between.

Enzymes are objects within cells that can bind and metabolize solutes. Note an enzyme is an object named for convenience. It does not represent actual metabolic enzymes. Rather, an enzyme maps to a portion of material within a cell that can influence metabolism of the solute’s counterparts within a simulation cycle. So doing is a necessary consequence of adherence to our strong parsimony guideline, which includes using single object types as placeholders for what in the future may be a set of distinctly different objects. Like solutes, enzymes have a number of properties, including enzyme type. In the ISHC, there is a single type of enzyme that interacts with all bindable solutes. In this iteration of the ISL, there are four enzyme types: apap-enzyme, ant-enzyme, czn-enzyme, and nonspecific. Different enzyme types have type-specific properties, including a list of which solute types can bind to that enzyme type (see Table 3). Different cell types can also contain (“express”) different enzyme types (expanded upon below). For simplicity, we specified enzyme types according to the corresponding drug. For example, apap-enzymes exclusively bind and metabolize apap objects. Nonspecific can bind all bindable solutes, but cannot metabolize them. While finer-grain knowledge of which P450 isoforms metabolize APAP, ANT, and CZN in both rats and humans is available [29,32–35], as implied above, parsimony dictates that we do not include that level of granularity until validation targets cannot be achieved without doing so. Similarly, [21] measured levels of CYP3A2 and CYP2C11 separately; however, relative changes after LPS pretreatment were extremely similar for both isozymes. Thus, including two distinct enzyme types for ant experiments was unnecessary.

**Table 3:** Enzyme-specific parameter values for validating experiments.

Enzyme type	*expressingCellTypes*	*acceptedSolutes*	*metabolic*	*downRegulated*	*pRemove*	*bindExponent*
apap-enzyme	hepatocyte	apap	TRUE	TRUE	0.01	1.0
ant-enzyme	hepatocyte	ant	TRUE	TRUE	0.025	2.0
czn-enzyme	hepatocyte	czn	TRUE	TRUE	0.02	1.5
nonspecific	Kupffer cell, endothelial cell	apap, ant, czn	FALSE	FALSE	N/A	1.0

“N/A” values denote that the parameter is not relevant in that simulation (e.g. METABOLISM HANDLER parameters in CELLS without metabolizing ENZYMES).

Cells are agent objects that maintain state information and can contain enzymes and solutes. The three cell types used in simulations here are hepatocytes, endothelial cells (ISL only), and Kupffer cells. Hepatocytes contain enzymes. Endothelial cells contain only nonspecifics. Kupffer cells do not contain enzymes. Each type of cell contains various physiomimetic mechanism modules [12] that are executed once per simulation cycle in pseudo-random (simply random hereafter) order. When executed, mechanism modules act upon cell contents (solutes and enzymes) and other cell state information (see “ISL and ISHC mechanisms” for details).

### ISL and ISHC structure

Full details of ISL structure are provided elsewhere [13,14]. For this work, we plugged the cited lobule object into a simple body compartment (Fig 1C). Together, they map to a rat. Body maps to plasma plus all other drug-accessible tissues; lobule maps to the rat liver. Injected solutes (i.e. lps and/or drug) are added to body during the simulation, and body transfers a fraction of its solutes to the portal vein of the lobule each simulation cycle. In silico measurements of solute are sampled from body. Lobule is a directed graph—or sinusoid network—of interconnected nodes and edges. Each node is a sinusoid segment, which maps to a portion of sinusoid. Edges map to direction of blood flow, from the portal vein to the central vein. Once transferred from body to portal vein, solutes percolate through sinusoid segments and the edges connecting them. Surviving solutes reach central vein and return to body. Sinusoid segments contain an innermost core, an outermost bile canal, and concentric, cylindrical spaces. Cellular spaces contain grid points that can contain at most one cell. Acellular spaces can only contain solutes. Moving radially outward from core, solutes may enter blood-cell interface (acellular), endothelial space (contains endothelial cells and Kupffer cells), space of Disse (acellular), hepatocyte space (contains hepatocytes), and bile canal (acellular).

ISHC structure is composed of two stacked, rectangular grids (Fig 1B), each mapping to different in vitro spaces [12]. Cell space maps to the monolayer of cells; each grid point contains at most one cell (hepatocyte or Kupffer cell). Media space maps to culture media. Both spaces may contain solutes, which can move between spaces or laterally within a space. To account for the much greater volume in culture media compared to the cell monolayer, between-grid movement is asymmetrical: a solute can only move “up” from cell space to media space with probability *pExitCell*; a solute moving “down” uses the parameter *pExitMedia*, which is typically much smaller than *pExitCell*. Injected solutes are randomly assigned to media space and cell space grid points. Solute measurements are sampled from the entire system.

### ISL and ISHC mechanisms

Logic governing ISL and ISHC mechanism modules is outlined below, and flowcharts of mechanism logic are illustrated in S1 Fig. There are five mechanism modules used by cells in both the ISL and ISHC for current use cases. Mechanism names include the suffix handler to emphasize the fact that they are modules that directly act upon model components and their state information. In fact, outside of the function of the mechanism modules, solutes do nothing other than percolate through spaces, and enzymes do nothing other than exist within cells. Each mechanism is probabilistic in nature. When an event occurs with probability *p*, a random draw from the standard uniform distribution, *U*[0,1), determines whether the event occurs. If the random draw is less than *p*, the event occurs. Event execution is handled using a scheduler that conducts the timing and ordering of events.

Binding handler maps to both specific and nonspecific binding processes in the cell. This mechanism module is present in all hepatocytes and endothelial cells. When the mechanism is executed, each unbound solute inside the cell has a chance to bind to an unbound enzyme. With probability *pBind*, a solute binds to an unbound enzyme. While a solute is bound, it cannot leave that cell, bind to another enzyme, or be removed via degradation; however, it can be metabolized (see below). While an enzyme is bound, it cannot be removed (e.g. via down-regulation) or bind another solute. Upon binding, the solute is scheduled to be released (unbound) from the enzyme after *bindCycles* simulation cycles.

Metabolism handler maps to metabolism via P450 enzymes; it is unique to hepatocytes. When executed, there is a chance for each bound solute to be metabolized with probability *pMetabolize*. When a solute is metabolized, two events occur. 1) The enzyme that metabolized the solute is no longer bound and is thus free to bind and metabolize again. 2) The metabolized solute is removed from the system and replaced with its appropriate metabolite. The solute’s state information specifies which metabolite type is produced. If the solute specifies multiple metabolite types, one is randomly selected. If the solute does not specify a metabolite product, it is simply removed from the system and is not replaced.

A different value of *pMetabolize* is specified for each solute type, allowing for different types of solutes to be metabolized at different rates. In simulations here, a particular solute type has only one corresponding enzyme type that can metabolize it. However, some use cases may require multiple enzyme types that can metabolize a particular solute type. In such cases, a different value of *pMetabolize* can be specified for each pairwise combination of solute and enzyme types. Thus, metabolism can be differentially controlled by individual solute and enzyme types.

Inflammation handler maps to cytokine production in response to an inflammatory stimulus like LPS; it is unique to Kupffer cells. When executed, the mechanism has a chance to produce a cytokine; if so, the cytokine is added to the Kupffer cell. To determine whether to produce a cytokine, it first counts how many inflammatory stimuli are in the Kupffer cell. An inflammatory stimulus is any solute for which the Boolean property *inflammatory* is true. The only inflammatory stimulus is lps. If the number of inflammatory stimuli exceeds the value of the parameter *inflammatoryThreshold*, there is a chance to produce a cytokine. With probability *pCytokine*, a cytokine is produced. The value of *pCytokine* is variable, determined by several factors:

$$pCyotkine=1-\exp\left(-\frac{\#ɪɴꜰʟᴀᴍᴍᴀᴛᴏʀʏ\;ꜱᴛɪᴍᴜʟɪ-inflammatoryThreshold}{cytokineExponent}\right)$$

Thus, *cytokineExponent* controls the degree to which increasing stimulus causes cytokine formation. Lastly, to prevent excess cytokine formation in the presence of significant inflammatory stimulus, cytokine cannot be created if there are already more than *cytokineThreshold* cytokines in the cell.

The above equation is an example of a biomimetic rule. We lack sufficient detailed knowledge to describe exactly how a Kupffer cell in a particular lobule location determines whether to produce cytokine. The equation is a placeholder for yet to be specified fine-grain mechanisms. A risk of relying on such rules is committing inscription error, the logical fallacy of assuming the conclusion and programming in (consciously or not) aspects of the result we expect to see [36]. Cognizant of inscription error, we were careful not to explicitly encode a sigmoidal dose-response into inflammation handler. We chose the above equation to mimic a Poisson distribution in which the probability of at least one binding event occurring within a simulation cycle depends on the extent to which the number of inflammatory stimuli exceeds a threshold value. The ability to generate a sigmoidal dose-response curve arises from a complex interplay among multiple analog mechanisms, facilitated by flexibility in the parameters *inflammatoryThreshold* and *cytokineExponent*.

Down-regulation handler maps to P450 down-regulation processes in response to a cytokine signal; it is unique to hepatocytes. This mechanism is more complex and can follow one of two pathways: when executed, it has a chance to either 1) remove an enzyme (in response to sufficient cytokine) or 2) create an enzyme (when cytokine is not present). Enzyme removal maps to P450 down-regulation; enzyme creation maps to the gradual return to basal P450 levels. Importantly, each hepatocyte contains a separate instance of down-regulation handler for each down-regulatable enzyme type (call it *T*) it contains. In the descriptions that follow, each instance operates only on enzymes of type *T*. The instances operate independently.

When cytokine is present in the hepatocyte, an enzyme has a chance to be removed. For each cytokine, the probability to do so is *pRemove*. However, the enzyme is not removed immediately; instead, enzyme removal is scheduled to occur after *delay* simulation cycles. Until then, the enzyme is marked for removal but is not actually removed. The more cytokines are present, the more chances there are to schedule enzyme removal; however, to prevent abrupt changes in the number of enzymes, at most one enzyme may be scheduled for removal each simulation cycle. Further, if additional enzymes are scheduled for removal before the previously scheduled enzyme is actually removed, their delay is added to the tail end of the currently remaining delay. Thus, when cytokine levels are sufficiently high, there is a growing “queue” of scheduled enzyme removal events. Including the *delay* parameter and removal queue was necessary to prevent rapid enzyme down-regulation over the course of very few simulation cycles.

Alternatively, enzymes may be created when there are no more cytokines in the hepatocyte and after the removal queue has cleared. Further, this pathway can only occur when the number of enzymes is less than the number at the start of the simulation. Thus, enzymes can gradually return to their original (basal) levels. If the three conditions are met—no cytokines, empty queue, less than basal enzyme—an enzyme may be created with probability *pReplenish*.

We designed down-regulation handler to be sufficiently general so that, when needed, we can include specific subtypes of cytokines (or other solutes) that can specifically and differentially down-regulate different sets of enzyme types. Currently, each enzyme type can have a different value for *pRemove*. In the future, as mechanisms for individual cytokine types are teased out, *pRemove* can be specific to a particular pair of enzyme and cytokine type. In other words, parameters like *pRemove* can take on pairwise values: e.g. *pRemove*_*i*,*j*_, where *i* is a specific enzyme type and *j* is a specific cytokine type.

The final mechanism, degradation handler, maps to non-P450 degradation of compounds; it is in all cells. The mechanism is simple: when executed, each unbound solute can, with probability *pDegrade*, be “degraded” (removed from the system). There is no limit to the number of solutes that can be degraded each simulation cycle via this mechanism. Solutes that do not specify a value for *pDegrade* cannot be degraded.

Mappings between ISL/ISHC mechanism parameters (like *pMetabolize*) and kinetic, thermodynamic, and/or pharmacological properties exist, but need not be one-to-one. Quantitative mappings can be made via methods including linear regression, artificial neural network, or fuzzy clustering algorithms. Indeed, these methods have each been shown to successfully predict ISL parameter values for several drugs, which, when plugged into an ISL and tested, produce simulated hepatic outflow profiles that closely match wet-lab data [37].

### Enzyme mechanism falsification and generalization

The enzyme ontology and related binding and metabolism mechanisms were falsified by the observation that even a dramatic reduction in the number of enzymes does not significantly affect solute clearance. The explanation is that the probabilities of binding events—and therefore metabolism events—is neither a function of the number of enzymes nor the number of solutes that are already bound (provided there is at least one unbound enzyme). There are two exceptional cases: 1) the case in which all enzymes are bound and 2) the subset of this case in which there are zero enzymes. In both exceptional cases, the probability of a binding event drops to zero (S2A Fig). These cases were found to be rare or nonexistent except under extreme parameter settings. Thus, within typical parameter settings, effective binding probability is unaffected by changes in the number of enzyme (S2C Fig). The result is that a change, such as simulating P450 down-regulation, has no significant effect on solute clearance, thereby falsifying those earlier mechanisms. Hereafter, whenever necessary to avoid ambiguity between enzymes used earlier and those described below, we refer to the former as generation 1 enzyme (G1-enzyme) and refer to the latter as generation 2 enzyme (G2-enzyme).

Achieving validation targets like those drawn from P450 down-regulation required a finer-grained, more generalized enzyme ontology and mechanisms for binding and metabolism. We implemented the following refinements.

### enzyme-specific properties

Whereas all G1-enzymes were controlled by the same set of parameters, G2-enzymes are dynamic data structures that have enzyme-specific parameters and maintain enzyme-specific state information. Among important parameters is enzyme type, which allows other components to recognize and interact differently with different types of enzyme. Each G2-enzyme is assigned a type (e.g. “Phase 1”), which maps to some set of xenobiotic-metabolizing enzymes. This allows different model components to recognize and interact with enzymes differently based on type. G2-enzymes can also have type-specific properties. For example, each enzyme type contains a list of which solute types can bind enzymes of that type. Other parameters include Booleans controlling whether that enzyme can metabolize solute (as opposed to binding only), is induced by drug, is down-regulated by cytokine, etc.

Each cell contains G2-enzymes of each type required by the current use case. Different cell types within a simulation can contain (or “express”) a different—possibly overlapping—set of enzyme types, which are specified offline via a parameter file (see Table 3). In ISL simulations here, hepatocytes contain all four enzyme types (apap-enzyme, ant-enzyme, czn-enzyme, and nonspecific), and endothelial cells contain only nonspecific.

### Parameter-controlled binding probability

G2-enzymes can bind and metabolize solutes similarly to G1-enzymes; however, binding need not be controlled by a single constant parameter, *pBind*. Rather, the number of unbound enzymes of a given type in a given cell determines the probability of a binding event. The probability function used is controlled by a whole-model parameter, *bindingMode*. Currently, there are two binding modes: stepwise and variable (illustrated in S2 Fig). Stepwise mode is intended to recapitulate the previous G1-enzyme mechanism behaviors, used primarily for verification purposes. Using the stepwise mode:

$$P(\text{binding event})=\left\{ \begin{array}{ccc} pBind & & \#unbound>0\\ 0 & & \#unbound=0 \end{array}\right.$$

where *#unbound* is the total number of unbound enzymes. Thus, the binding probability is either zero (when all enzymes are bound) or *pBind* (when at least one enzyme is unbound) (S2C Fig). Using the variable mode:

$$P(\text{binding event})=pBind∙{\left(-\frac{\#bound-total}{{total}_{\text{initial}}}\right)}^{bindExponent}$$

where *#bound* is the total number of bound enzymes, *total* is the total number of enzymes (bound or unbound), and *total*_initial_ is the total number of enzymes at the start of the simulation. Thus, the binding probability decreases as more enzymes bind (moving down the line) and/or as enzymes are removed from the system (shifting the curve downward) (S2D Fig). The parameter *bindExponent* controls the nonlinearity of this effect (S2B Fig). The probability reaches zero when all enzymes are bound (i.e. the number of bound enzymes reaches the current total number of enzymes). Further, the *y*-intercept (the probability of binding when there are no bound enzymes) is scaled by *total* normalized by *total*_initial_. Thus, changes in the total number of enzymes are two-fold (S2D Fig): it changes both the probability of binding and the maximum number of solutes that can be bound to the enzyme (*x*-intercept). Because all metabolism events are preceded by a binding event, changes in binding probability will directly affect metabolism and clearance.

G2-Enzymes are designed to be inherently tunable [38]. If a future use case requires addition of non-P450 enzymes or further delineation into (say) specific P450 isoforms, the G2-enzyme structure can still be used. On the other hand, if a future use case does not require this level of granularity, the G1-enzyme mechanisms can be restored by specifying only two enzyme types (enzyme and nonspecific) and setting the binding probability function to stepwise mode. In this way, the phenotype space of the G2-enzyme mechanism subsumes that of the G1-enzymes mechanism; that is, the new mechanism is a generalization of the earlier mechanism.

### Mechanism granularity and flexibility

It is well established that a particular drug may be metabolized by several enzymes, each of which may be differentially down-regulated (or perhaps up-regulated or unaffected) by different cytokines. Given that a G2-enzyme object does not map to a particular P450 isoform, why should we compare enzyme measurements to wet-lab data using a single, specific P450 isoform? A similar question can be asked of cytokines. The answer is two-fold. Firstly, the chosen validation data is not sufficiently detailed to extract differential effects of multiple specific P450 isoforms or specific cytokines. Following our strong parsimony guideline, analog mechanisms should not include that level of isoform- or cytokine-specific detail unless and until doing so is needed to achieve validation targets. For example, the validation data do not measure the regulation of more than one or two P450 isoforms. ([21] measures two isoforms, but they are down-regulated almost the same amount.) Thus, current use cases only require simulation and measurement of a single metabolizing enzyme type per drug. Secondly, the current use case is to develop flexible methods for simulating immune-mediated P450 down-regulation. Future use cases may include validating against differential isoform- and/or cytokine-specific data. In these cases, the ISL and ISHC have built-in capabilities to further delineate enzymes and cytokines using only changes in parameter values. Their differential effects on inflammation and P450 down-regulation can subsequently be specified using cytokine-specific parameters (e.g. *inflammatoryThreshold*) and pairwise cytokine-enzyme parameters (e.g. *pRemove*), respectively.

### Modularity and integration

We modularized the analog components and mechanisms using methods outlined in [12]. Modularization facilitates reusing and integrating components among different models. For example, immune system components (namely Kupffer cell) were initially developed as part of the ISHC. Since components were modularized, they were easily shared with the ISL with minimal code refactoring.

### Selecting parameter values

Where applicable, we began with parameter values used to achieve validation targets in previous ISL and ISHC publications (e.g. [12,13]). For parameters introduced in this work, we began with modest values: e.g. 0.5 for probabilistic parameters like *pRemove* with range [0,1], 1 for threshold parameters like *inflammatoryStimuli* based on number of objects, and 1.0 for weighting parameters like *cytokineExponent*. From there, parameter value options are evaluated iteratively, following Steps 5–7 of the IRP (see Fig 1A). Simultaneous, small changes (e.g. 5–10%) in several parameter values can offset each other and may produce no detectable change in a measured phenomenon. Thus, conventional linear sensitivity studies are less informative and meaningful than complete location changes in analog parameter space. When needed, we use batch parameter space sampling (as in [39,40]) to identify small subsets of parameter values that are most influential for particular attributes. We also often rely on heuristics and trial-and-error to arrive at validating parameterizations. For the exploratory studies herein, we only present one validating parameter set for each experiment (see Table 1). Narrative explanations for parameter value choices are provided at the end of each experiment in Results.

## Results

A full list of parameters descriptions and their values used for validation experiments is provided in Table 1. Some parameters are specific to the solute or enzyme types used in each simulation; these parameter values are provided in Tables 2 and 3, respectively. Validation targets and associated validation data are summarized in Table 4.

**Table 4:** Validation targets achieved for immune-mediated P450 down-regulation attributes.

Targeted attribute	Validation data	Similarity criteria
Kupffer cells produce cytokine upon LPS stimulus in vitro (Fig 2A).	Dose-response curve between LPS dose and TNF-α response using an in vitro Kupffer cell culture [23].	In silico values fall within ± 1 standard deviation of wet-lab values.
Cytokines down-regulate hepatic P450 levels in vitro (Fig 2B).	Time-course drop in P450 levels after IL-1 stimulus using an in vitro hepatocyte culture [24].	In silico values fall within ± 1 standard deviation of wet-lab values.
LPS reduces APAP clearance in rats (Figs 3–5).	1) Disappearance curves and 2) half-life values with/without LPS pretreatment in rats [20].	1) >50% in silico values fall within ± 25% of wet-lab values. 2) In silico values fall within ± 1 standard deviation of wet-lab values.
LPS reduces ANT clearance (Figs 3–5) and CYP3A2/2C11 levels (Fig 2C) in rats.	1) Disappearance curves, 2) relative CYP3A2/2C11 levels, 3) half-life values, and 4) relative systemic clearance with/without LPS pretreatment in rats [21].	1) >50% in silico values fall within ± 25% of wet-lab values. 2) In silico values fall within ± 1 standard deviation of wet-lab values.
LPS reduces CZN clearance (Figs 3–5) and CYP2E1 levels (Fig 2C) in rats.	1) Disappearance curves, 2) CYP2E1 levels, 3) half-life values, and 4) relative intrinsic clearance with/without LPS pretreatment in rats [22].	1) >50% in silico values fall within ± 25% of wet-lab values. 2–4) In silico values fall within ± 1 standard deviation of wet-lab values.

### ISHC experiments

We first aimed to achieve a degree of validation for the first part of the pathway: an LPS stimulus causes Kupffer cell-mediated cytokine release. The validation data, derived from [23], is a dose-response curve between LPS dose and TNF-α response using an in vitro Kupffer cell culture. TNF-α was measured after 48-hr LPS treatment using LPS concentrations of 0, 0.1, 1, 10, and 1,000 ng/ml. To simulate this in vitro Kupffer cell culture, we instantiated an ISHC with the only cells being Kupffer cells (i.e. *kcDensity* = 1.0 and *hepDensity* = 0.0). We specified the analog-to-referent mapping of 700 lps objects to 1 ng/ml LPS, and 1 simulation cycle to 1 min. We arrived at this LPS mapping after several iterations, as it provided a consistent sigmoidal dose-response curve over a wide range of parameter values. The time mapping was the same as previous ISHC experiments [12]; it is intentionally coarse-grain, as current use cases do not require mimicking temporally fine-grain phenomena.

To mimic the dose-response curve between 0 and 1,000 ng/ml LPS, a dose ranging from 0 to 700,000 lps objects was injected at the start of the simulation. The total number of cytokines was measured after 2,880 simulation cycles (maps to 48 hr). The resulting values were used to construct a dose-response curve, normalized by the maximum obtained number of cytokines. We prespecified that the analog dose-response curve would be acceptably similar to the referent if each in silico point falls within ± 1 standard deviation of the corresponding wet-lab value. In accordance with the IRP, we sampled ISHC parameter space until similarity criteria were achieved. The resulting validated dose-response curve is shown in Fig 2A.

When selecting parameter values and the LPS analog-to-referent mapping for this experiment, the resulting dose-response curve followed a sigmoidal shape for all tested parameter values. Thus, we first arrived at a robust choice for the LPS analog-to-referent mapping. The LPS mapping was chosen as a balance between granularity and computational efficiency. It is reasonable to strike that balance because both ISL and ISHC are analogies, not one-to-one models of their referents. When the mapping was too large (e.g. 1000 lps objects maps to 1 ng/ml LPS), large doses (over one million lps objects) significantly increased computational costs. When the mapping was too small (e.g. 100 lps objects maps to 1 ng/ml LPS), the smallest non-zero dose (mapping to 0.1 ng/ml LPS) consisted of only 10 lps objects (in the presence of 625 grid points in cell space), which was deemed too coarse-grain to provide meaningful insight. We arrived at the mapping of 700 lps objects to 1 ng/ml LPS after several iterations, then proceeded to test selected parameter values. The challenge in specifying parameter values was to control the microstructure of the resulting sigmoidal curve (e.g. the slope of the inflection point). In this case, *inflammatoryThreshold* was a sensitive parameter; it effectively controlled the dose at which the cytokine response begins to sharply increase. The value of *pDegrade* for cytokine was also sensitive; when too small (e.g. 0.001), the slope of the inflection point was too shallow; when too large (e.g. 0.1), the inflection point would shift too far to the right.

We then aimed to achieve a degree of validation for the second part of the pathway: an increase in cytokines results in hepatic P450 down-regulation. The targeted attribute was data from an in vitro hepatocyte culture: specifically, time-course P450 levels after IL-1 stimulus [24]. We mimicked the in vitro hepatocyte culture by instantiating an ISHC with only hepatocytes (i.e. *kcDensity* = 0.0 and *hepDensity* = 1.0). A dose of 2,000 cytokines was injected at the start of the simulation. The total number of G1-enzymes was measured at intervals mapping to corresponding wet-lab points (0, 12, and 24 hr), plotted against time, and normalized by the starting number of G1-enzymes. Similarity criteria stipulated that each in silico time measurement fall within ± 1 standard deviation of the corresponding wet-lab value. Fig 2B illustrates the time-course plot after identifying parameter values that achieved validation.

Despite down-regulation handler being a finer grain mechanism, specifying parameter values for Fig 2B was straightforward. Parameter value choices were influenced by the following deduction: given enough time, the simulation would eventually return to basal levels of enzyme. This is because the cytokine signal would eventually die due to degradation handler, and enzymes will eventually be replenished (provided *pReplenish* is greater than zero). That scenario is consistent with what one would expect physiologically. Thus, a moderate value for *delay* (mapping to 30 minutes) and small value for *pReplenish* (0.007) produced the relatively slow time-course P450 down-regulation as found in the wet-lab validation data.

### Single-drug ISL experiments

The next three sets of validation targets were designed to achieve degrees of validation for the entire coarse-grain pathway: an LPS stimulus results in hepatic P450 down-regulation and downstream changes in measures of drug clearance. We drew validation targets from three wet-lab experiments, each of which followed a similar experimental protocol but used a different drug and measure of drug clearance. Each experiment pretreated rats with LPS (experimental) or saline (control) for 24 hr before injecting a drug. Clearance was measured at prespecified time points following drug injection. Enzyme measures were taken after the 24-hr pretreatment. All experiments measured disappearance curves from blood (APAP) or plasma (ANT, CZN) for their respective drug. In addition, [20] provide APAP half-life values; [21] measure ANT half-life and systemic clearance (CL_sys_) and CYP3A2/CYP2C11 levels; [22] measure CZN half-life and create a scatterplot of CZN intrinsic clearance versus CYP2E1 levels. Similarity criteria were prespecified as follows. For drug disappearance curves, at least 50% of in silico values must fall within ± 25% of the corresponding wet-lab value. For enzyme measurements, in silico values must fall within ± 1 standard deviation of the wet-lab value. For all clearance measures except ANT half-life, the in silico value must fall within ± 1 standard deviation of the corresponding wet-lab value. Standard deviation for ANT half-life was neither given nor able to be determined from the data given, so we specified the similarity criteria that in silico values fall within ± 10% of the corresponding wet-lab value.

Simulating whole rat experiments required switching from experimenting on an ISHC to an ISL. We first instantiated an ISL for each of the six experiments: two treatment groups (control and LPS) for each of three drugs. For ISL simulations, we specified the analog-to-referent mapping of 1 simulation cycle to 1 sec, consistent with previous ISL experiments [37]. This constitutes a 60-fold finer temporal resolution than the ISHC mapping of 1 simulation cycle to 1 min. Thus, the ISL is intended to be a temporally finer-grained analog, as required to capture hepatic transit times on the order of seconds. (Note we did not need analog-to-referent mappings for drug or enzyme concentrations because measurements were normalized.) At the start of each simulation in the LPS group, an initial dose of 125,000 lps objects was injected into body. After 86,400 simulation cycles (maps to 24 hr), a second dose of 125,000 drug objects (apap, ant, or czn) was injected into body. At this point we also measured the total number of enzymes of the type corresponding to the injected drug. At time points corresponding to the respective wet-lab data, we measured the number of drug objects in body.

For control group simulations, we bypassed the 86,400-simulation cycle phase. So doing spared computational costs without altering simulation results, because without lps there are no ISL mechanisms that could alter enzyme levels or affect drug clearance; further, these simulations do not require “priming” from initialization. Thus, there was only a single dose of 125,000 drug objects at the start of each control group simulation. After drug injection, control and LPS group experiments operated identically.

Various parameter value combinations were evaluated for each experiment until validation targets were achieved. We imposed several constraints when selecting those parameters. Namely, we ensured that whole-model parameter values (which are neither solute- nor enzyme-specific) could not change among the six experiments. Furthermore, drug and enzyme properties could not change between control and LPS experiments of the same drug type. The reasoning behind these constraints was to mimic experimenting on the same (or similar) rats. For experiments involving a particular drug (say apap), the drug-specific parameter values of other drugs (i.e. ant and czn) were still included in the parameter set, but their dose was set to zero. Similarly, all three enzyme types are included in apap simulations, though ant-enzymes and czn-enzymes do not affect apap results. Thus, the only parameter values that actually changed between simulation experiments were in silico experiment design parameters (i.e. those having no biological counterpart): those specifying dose (i.e. whether or not to inject lps; which drug to inject), experiment duration (i.e. how many simulation cycles), and measurements (i.e. when to take measurements). As a consequence of the above constraints, validation is properly limited to those cases in which each experiment achieved validation targets using a common set of in silico experiment design parameters.

Meeting the above set of strict validation targets required several rounds of iterative refinement. The most significant refinement was the need to generalize enzyme mechanisms, outlined in Methods. While ISHC experiments achieved validation targets using G1-enzymes and associated mechanisms, achieving ISL validation targets required G2-enzymes. Additional refinements are detailed in Discussion.

**Fig 3:**
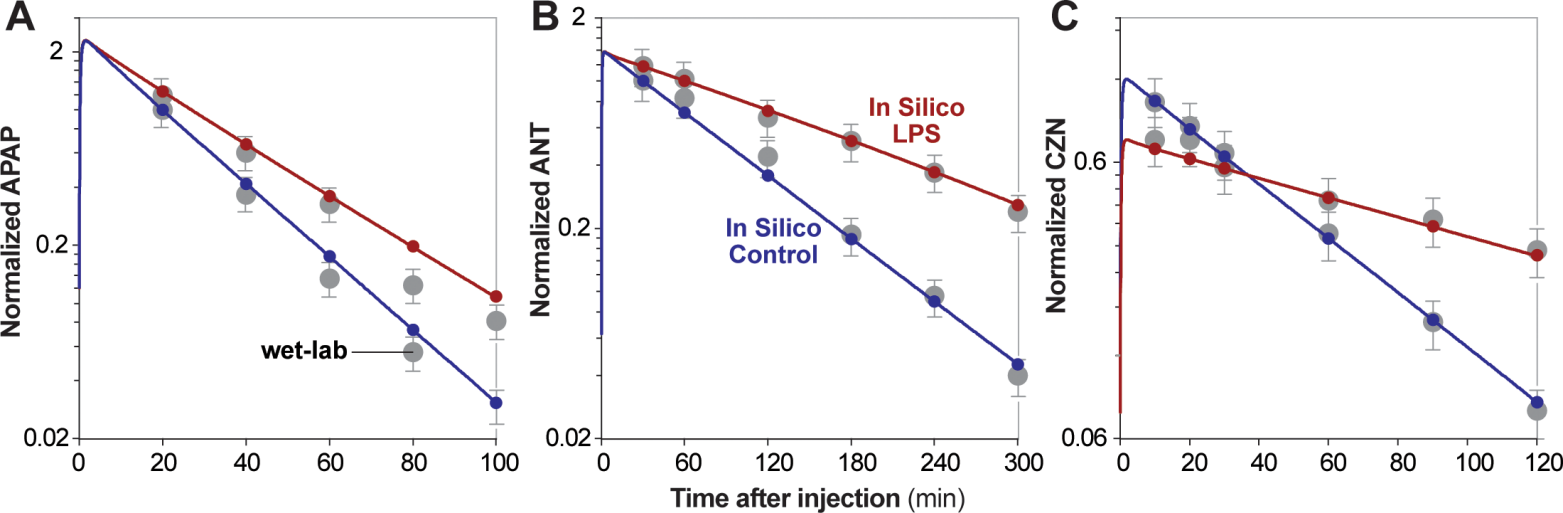
Wet-lab and in silico normalized drug disappearance curves. **A.** APAP [20]; **B.** ANT [21]; **C.** CZN [22]. Red/blue circles: in silico averages of 16 Monte Carlo trials. Gray circles: wet-lab averages. Red/blue lines: additional in silico values between wet-lab time points. The initial spike in drug corresponds to the administered dose. All drug values are normalized by the control value at the first time point. Error bars: ± 25% of the wet-lab value (the similarity criteria).

**Fig 4:**
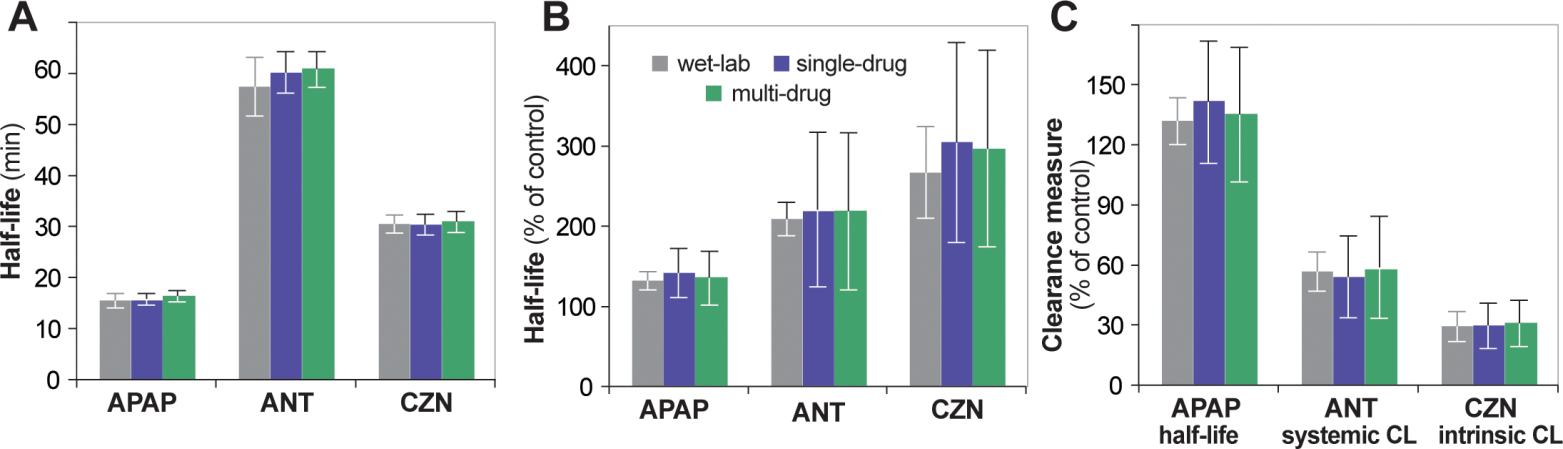
Wet-lab and in silico measures of drug clearance. **A.** Wet-lab and in silico half-life measures without LPS pretreatment (control). **B.** Wet-lab and in silico half-life given 24 hr LPS pretreatment, relative to control. **C.** Wet-lab and in silico clearance measures given 24 hr LPS pretreatment, relative to control. Error bars: standard deviation. Wet-lab values are from [20] (APAP), [21] (ANT), and [22] (CZN).

**Fig 5:**
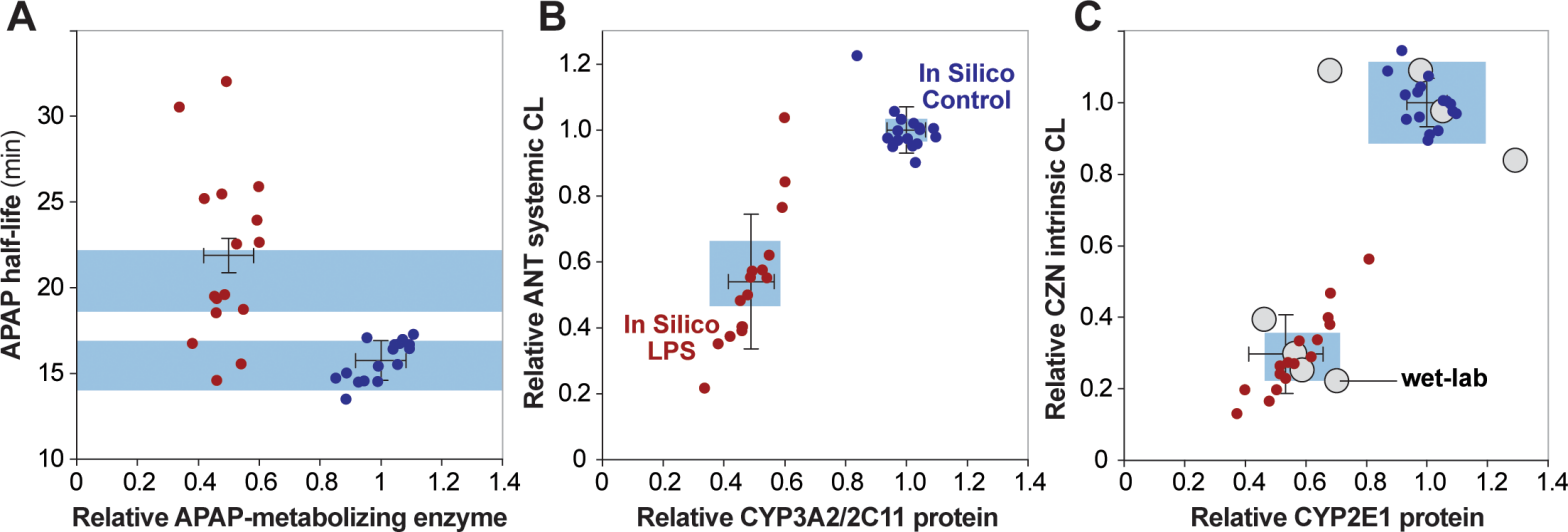
Scatterplots between enzyme measurements and clearance measurements for both control and LPS experiments. **A.** APAP [20]; **B.** ANT [21]; **C.** CZN [22]. Gray circles: wet-lab data points (when provided). Red/blue circles: in silico data points. Error bars: in silico standard deviation, extending from the mean of 16 Monte Carlo trials. Blue box: area of acceptable similarity (± 1 standard deviation of wet-lab value). Since [20] did not provide enzyme data, there is no associated validation target (**A**). Only [22] provided values for individual wet-lab trials (**C**).

The following figures recapitulate the selected wet-lab data using in silico results from validating experiments. Fig 3 shows the disappearance curves with wet-lab data overlaid. Sixty percent of values fall within acceptable similarity (± 25% of the wet-lab value) for APAP data; 100% of values fall within acceptable similarity for ANT and CZN data. Fig 2C shows the relative change in P450 levels after 24 hr LPS pretreatment. While [20] did not provide P450 data (and thus no validation targets can be drawn for apap experiments), in silico values for ant and czn experiments fall within the prespecified similarity criteria of ± 1 standard deviation of the wet-lab value. In fact, they fall within a more stringent ± 0.5 standard deviation similarity criteria. Fig 4 shows the half-lives for control experiments, relative half-lives for LPS experiments, and relative clearance measures (half-life, systemic clearance, and intrinsic clearance for APAP, ANT, and CZN, respectively) alongside validation data. All values fall within acceptable similarity (± 1 standard deviation of the wet-lab value). Lastly, Fig 5 shows scatterplots of relative enzyme versus clearance measures, with validation data overlaid.

Whereas ISHC experiments focused on small parts of the P450 down-regulation pathway—and thus selecting parameter values required focus on the one or two relevant mechanisms—the process for ISL experiments required attention to all mechanisms. For example, changes in inflammation handler parameter values affect cytokine levels, which directly affects downstream down-regulation handler behavior even if its parameters do not change. We first focused on inflammation handler because it contains no solute- or enzyme-specific parameters. Most notably, *inflammatoryThreshold* was chosen higher than the ISHC value (see Table 1); this was necessary to avoid excessive cytokine levels. Down-regulation handler required significant parameter changes compared to the ISHC. The down-regulation handler parameters are all sensitive to time: an enzyme removal or replenish event may be scheduled each simulation cycle. Thus, it is not surprising that the ISL—with a 60-fold finer grain temporal mapping than the ISHC—required smaller values for *pRemove* and *pReplenish* and a larger value for *delay* compared to the ISHC. The simulation must eventually return to basal levels of enzyme if left running indefinitely. However, if down-regulation were too sensitive (e.g. *pRemove* too high or cytokine’s *pDegrade* too low), enzyme levels could reach zero for long periods of time before replenishing. Thus, selection of parameter values often revolved around achieving sustained, but not excessive, levels of P450 down-regulation.

### Multi-drug ISL experiments

Lastly, we repeated both control and LPS experiments for a new use case in which all three drugs are co-administered. Wet-lab validation data for such co-administration experiments in the context of P450 down-regulation are unavailable. However, in silico multi-drug experiments represent an important use case for several reasons. 1) Multi-drug simulations stand as challengeable predictions of hypothetical wet-lab co-administration experiment results. Given new wet-lab co-administration data that falsifies these predictions, we can then hypothesize model refinements in accordance with the IRP. Refinements may include analog mechanisms of drug-drug interactions; however, details of the path to revision would depend on the nature of the results. 2) We demonstrate a proof-of-concept that the ISL can be made robust to changes in analog drug, and thus able to support co-simulation of any number of drug types. Extensibility to multiple drug types is essential to support future simulations designed to better understand and anticipate drug-drug interactions. 3) We demonstrate that all three drug types can simultaneously achieve validation targets using a single ISL parameter set. Simultaneous validation is significant because, if achieved, we can then conclude that the drug- and enzyme-specific parameterizations are sufficient to span the mechanistic changes needed to generate patterns in drug clearance and P450 down-regulation. In other words, values for whole-model parameters that are neither drug- nor enzyme-specific (e.g. *hepDensity*) need not be specified independently for each drug type. 4) Most importantly, failure to achieve simultaneous validation could be taken as evidence that hypotheses and several aspects of the ISL are falsified.

Notably, for this work, multi-drug experiments were not intended to explore or mimic drug-drug interactions. However, we also did not expect identical results between single- and multi-drug simulations because the presence of additional drug objects (even without the inclusion of explicit drug-drug interactions) may still indirectly affect ISL mechanisms. For example, the flow of solutes depends on the total number of solute objects, so multi-drug experiments with a larger total number of solutes may lead to subtle changes in the cascading of events. We expect more pronounced differences between single- and multi-drug experiments when individual cytokines and P450 isoforms are made explicit and/or when we explicitly add mechanisms that handle drug-drug interactions.

While the three wet-lab validation studies administered different amounts of LPS stimulus, we assumed an equipotent stimulus. We do not expect this equipotent assumption to be biologically realistic; however, in the absence of P450 down-regulation wet-lab data spanning both single and multiple drug experiments, we needed to make some assumption to simulate multi-drug experiments. Thus, body was injected with 125,000 lps objects, similar to single-drug experiments. The second dose comprised 125,000 (for LPS experiments) or 62,500 (for control experiments) objects of each drug type (apap, ant, and czn). Thereafter, single- and multi-drug experiments operated identically.

The validation target for the multi-drug experiments was to simultaneously achieve all validation targets for the single-drug experiments. Besides the in silico experiment design parameters (those controlling dose, experiment duration, and measurements), parameter values were not changed between single- and multi-drug experiments. Notably, all multi-drug validation targets were achieved without changing parameterizations that achieved the single-drug validation targets. Figs 2C and 4 include values for the multi-drug experiments alongside single-drug results and wet-lab validation data.

## Discussion

We execute a series of virtual experiments that together achieve several quantitative validation targets involving immune system interactions in drug metabolism. The described mechanisms—those that finally achieved validation targets—are supportive of the mechanistic hypothesis that in response to LPS, Kupffer cells down-regulate P450 via inflammatory cytokines, thus leading to a reduction in metabolic capacity. Given the synthetic nature of the analogs, we can continue to iteratively refine them to test additional and/or alternative mechanistic hypotheses, validating an increasingly large set of targeted attributes along the way.

### Trajectory of model falsification and refinement

Falsification is the primary source of knowledge generation, integral to the scientific method itself [41]. Unfortunately, published computational models tend to describe a finished product without detailing—or even mentioning—the many rounds of falsification and revision along the way. Describing this trajectory of iterative falsification and refinement requires detailed annotations of simulation experiments (including unsuccessful ones) and paying special attention to how, when, and why a mechanism fails to achieve given validation targets. Having that information is essential to the scientific process because it is falsification that provides new knowledge: specifically, the current (falsified) mechanisms are flawed—they are not a good analogy of the referent biological mechanisms [42]. Following falsification, analog mechanisms (the hypothesis) can be refined. Focusing on falsification as a scientifically *productive* endeavor helps one adhere to parsimony, as it curbs the tendency to commit inscription error and/or to succumb to the urge to incorporate “everything we know” into a model.

We describe the trajectory of three demonstrative falsification and refinement efforts encountered with the P450 down-regulation validation targets, and commentate on any mechanistic insight gained from this new knowledge. 1) We described earlier the G1-enzyme mechanism falsification and the need for the finer-grain, generalized G2-enzyme (see “Enzyme mechanism falsification and generalization”). Validation required three changes: 1) enzyme concentration-dependent binding probability 2) enzyme type-specific properties, and 3) the inclusion of various enzyme types within a single simulation. Taken together, these changes suggest that differential properties of multiple groups of enzymes influence drug clearance events during inflammatory states. While this dependence might be expected, we anticipate providing additional insight when ISL experiments move toward explicitly modeling individual P450 isoforms.

2) The large drop in CZN clearance (drops to 29.2% of control value), coupled with a moderate drop in CZN enzyme levels (drops to 57.8% of control value), could not initially be reproduced in silico. Using original mechanisms, relative decreases in clearance values were always too weak compared to corresponding decreases in the number of enzymes. After falsification, we noted that the wet-lab validation experiment recorded a 1.69-fold increase in CZN volume of distribution after the addition of LPS. Thus, we posited a revision hypothesis that validation could be achieved by adding a coarse-grain mechanism for changes in volume of distribution. Recall that a fraction of solutes in body are transferred to lobule each simulation cycle. This fraction is equal to *sampleRatio* (a whole-model parameter between 0 and 1) multiplied by *sampleRatioFactor* (a solute-specific parameter). We implemented the following coarse-grain change. In LPS experiments, the size of the initial dose and the value of *sampleRatioFactor* for czn are reduced by a factor of *V*_*d*,*change*_, where *V*_*d*,*change*_ is the wet-lab fold change in volume of distribution. After implementing this revision, we achieved the original validation targets. Thus, accounting for changes in volume of distribution is necessary to mimic P450 down-regulation phenomena for some drugs. As new, finer-grained validation targets are considered, ISL experiments can flesh out mechanistic details driving changes in volume of distribution.

3) Initially, inflammation handler did not include *cytokineThreshold*, a parameter that “turns off” cytokine production if there is enough cytokine in the cell. However, this exclusion resulted in unchecked cytokine formation (and thus a non-sigmoidal dose-response curve) when the amount of inflammatory stimulus was sufficiently high. Since our validation experiments included large amounts of cytokine (up to 700,000 objects), that mechanism was falsified. We implemented the *cytokineThreshold* parameter, with which we found sets of parameter values that achieved validation targets. This cytokine threshold mechanism is coarse-grain. It may map to more complex cytokine-suppression mechanisms including feedback inhibition and suppressor of cytokine signaling proteins [43]. Given sufficient validation data, we can add finer grain mechanistic features by introducing new solute types (e.g. those that map to suppressor of cytokine signaling proteins) and behaviors in place of the existing cytokine threshold mechanism.

### Current model falsification

Reporting validation results without demonstrating subsequent falsification would provide an incomplete picture of the IRP. We describe current model falsification to address both the limitations and future direction of the ISHC and ISL. The current models are limited to the majority of P450s that are down-regulated by an inflammatory state; however, a small subset of P450s are actually induced [1]. Clearance data for drugs primarily metabolized by this subset is expected to falsify existing mechanisms. Given that scenario, we can iteratively refine mechanisms to simultaneously allow both inflammatory-induced P450 down-regulation and induction.

The analogs are also falsified by additional immune system mechanisms other than P450 down-regulation. For example, inflammatory states cause leukocyte recruitment and localization via chemokines secreted from sites of necrosis [44,45]. Achieving validation targets drawn from these phenomena would require in silico mechanisms for leukocyte circulation, recruitment, and extravasation, as well as their downstream effects on hepatotoxicity. As we continue expanding the set of targeted attributes to include more diverse yet still interconnected phenomena, we expect IRP cycles to continue improving explanatory mechanistic insight into immune system involvement in drug metabolism and toxicity.

## Supporting Information

S1 Fig Flowcharts of mechanism logic. *U*[0,1) represents a random probability draw from the standard uniform distribution.
(TIF)

S2 Fig Binding probabilities used by binding handler. E1 –E4 represent four enzymes. S1 –S5 represent five solutes. **A.** Binding probability using stepwise binding mode. **B.** Binding probability using variable binding mode. **C.** P450 up- or down-regulation causes the binding curve to shift right or left, respectively, which has no effect on binding probability in the yellow region (typical range of number of bound enzymes). **D.** P450 up- or down-regulation causes the binding curve to shift right or left, respectively, which affects binding probability within the yellow region.
(TIF)

## Abbreviations

APAP: acetaminophen
ANT: antipyrine
CZN: chlorzoxazone
P450: cytochrome P450
ISHC: in silico hepatocyte culture
ISL: in silico liver
IL: interleukin
IRP: iterative refinement protocol
LPS: lipopolysachharide
*U*[0,1): standard uniform distribution
CL_sys_: systemic clearance
TNF-α: tumor necrosis factor-α
