# Supplementary figures and images for: Virtual Experiments Enable Exploring and Challenging Explanatory Mechanisms of Immune-Mediated P450 Down-Regulation
Source: PLoS One. 2016 May 26;11(5):e0155855. doi: 10.1371/journal.pone.0155855 (PMC4881988; doi:10.1371/journal.pone.0155855)

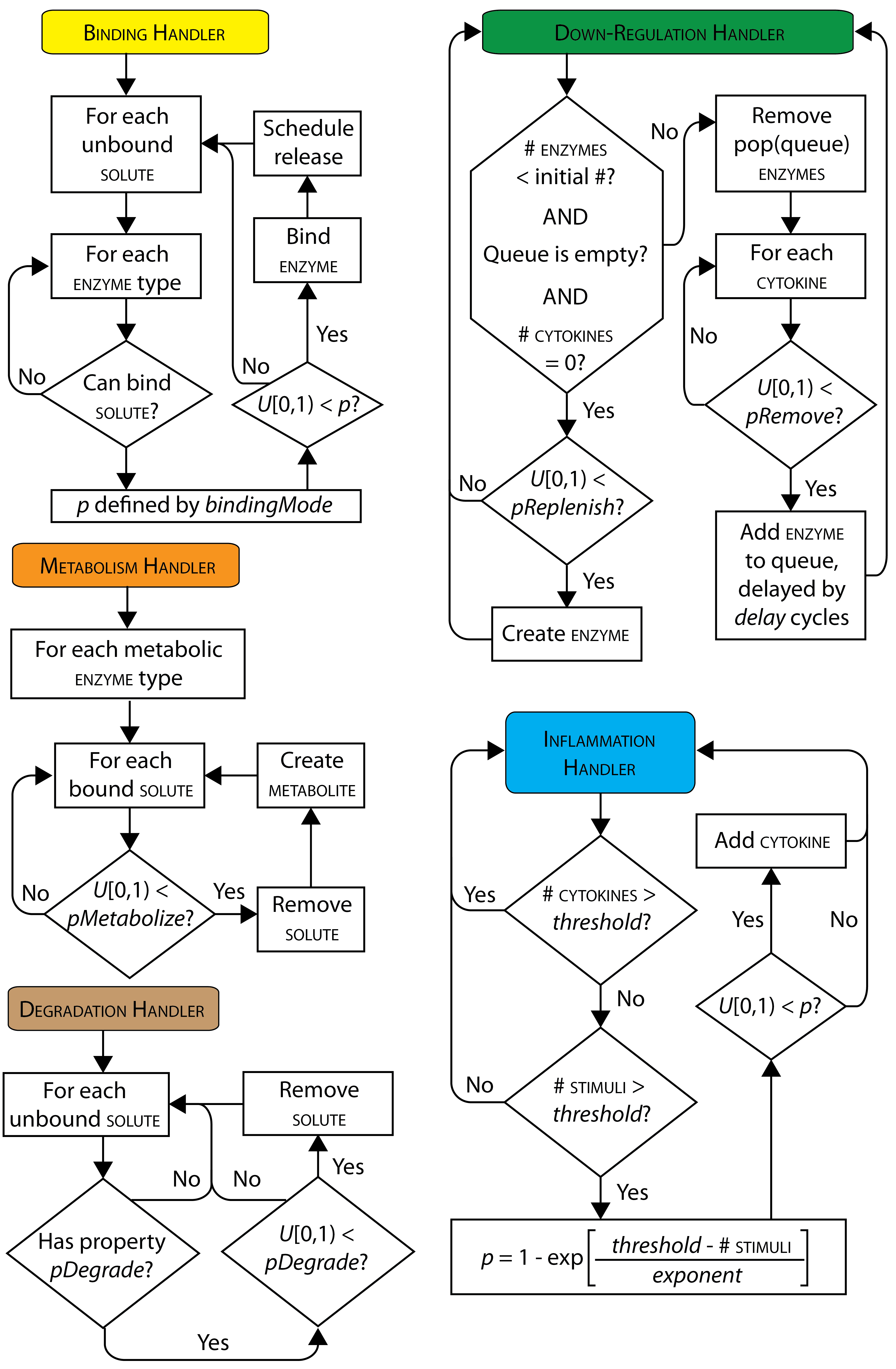

Supplement: S1 Fig — U[0,1) represents a random probability draw from the standard uniform distribution. (TIF) [file pone.0155855.s001.tif]

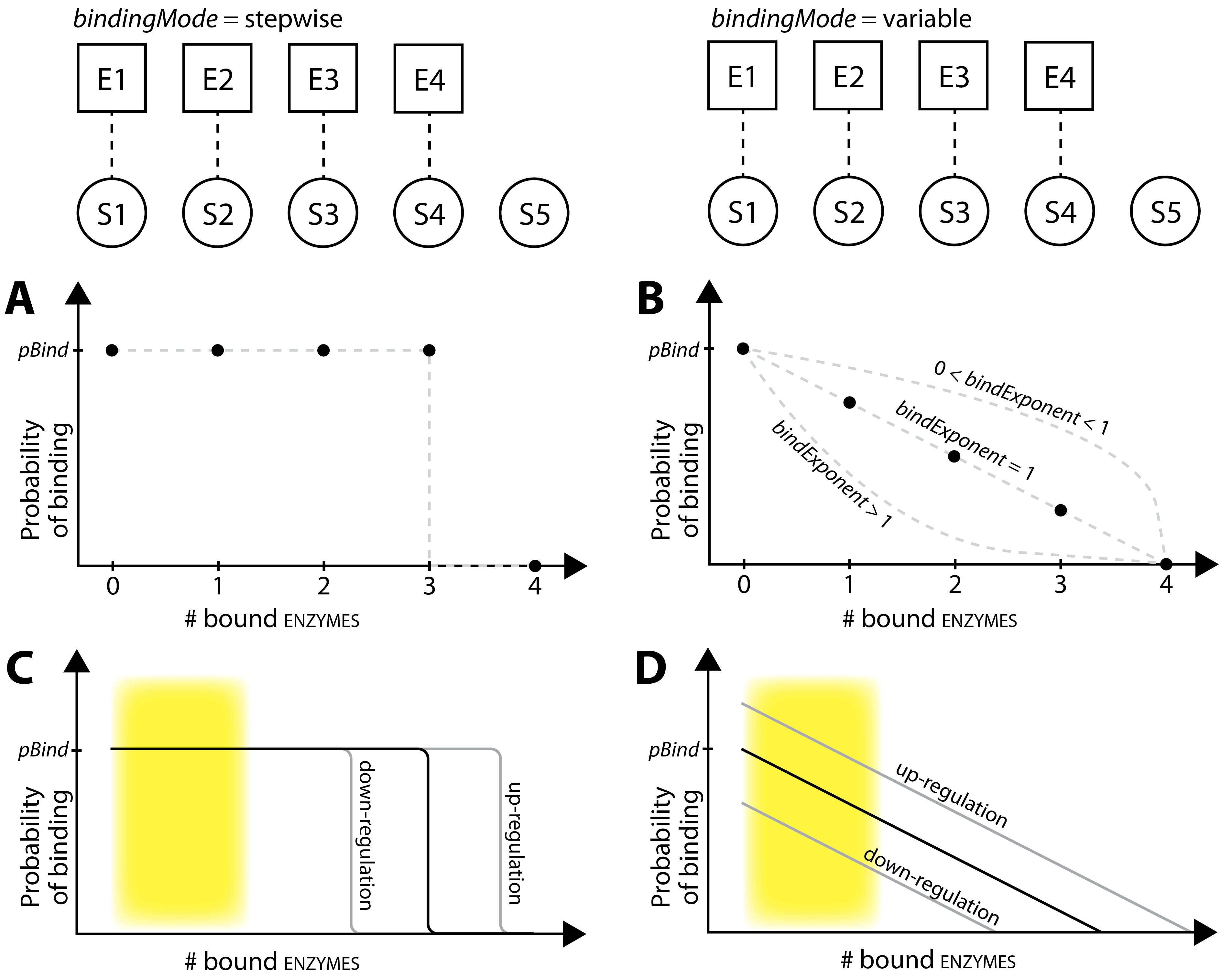

Supplement: S2 Fig — E1 –E4 represent four enzymes. S1 –S5 represent five solutes. A. Binding probability using stepwise binding mode. B. Binding probability using variable binding mode. C. P450 up- or down-regulation causes the binding curve to shift right or left, respectively, which has no effect on binding probability in the yellow region (typical range of number of bound enzymes). D. P450 up- or down-regulation causes the binding curve to shift right or left, respectively, which affects binding probability within the yellow region. (TIF) [file pone.0155855.s002.tif]
